# Investigation of airport sewage to detect importation of poliovirus, Poland, 2017 to 2020

**DOI:** 10.2807/1560-7917.ES.2022.27.24.2100674

**Published:** 2022-06-16

**Authors:** Arleta Krzysztoszek, Beata Gad, Sabine Diedrich, Sindy Böttcher, Magdalena Wieczorek

**Affiliations:** 1 Department of Virology, National Institute of Public Health NIH - National Institute of Research, Warsaw, Poland; 2 Regional Reference Laboratory for Poliomyelitis, Robert Koch Institute, Berlin, Germany

**Keywords:** environmental surveillance, poliovirus, Sabin-like polioviruses

## Abstract

**Background:**

Polioviruses are human pathogens which may easily be imported via travellers from endemic areas and countries where oral polio vaccine (OPV) is still routinely used to polio-free countries. Risk of reintroduction strictly depends on polio immunisation coverage. Sustaining a polio-free status requires strategies that allow rapid detection and control of potential poliovirus reintroductions.

**Aim:**

The aim of this study was to apply environmental surveillance at an international airport in Poland to estimate the probability of poliovirus importation via air transport.

**Methods:**

Between 2017 and 2020, we collected 142 sewage samples at Warsaw Airport. After sewage concentration, virus was isolated in susceptible cell cultures. Poliovirus isolates were characterised by intratypic differentiation and sequencing.

**Results:**

Seven samples were positive for polioviruses. All isolates were characterised as Sabin-like polioviruses type 3 (SL-3). No wild or vaccine-derived polioviruses were found. The number of mutations accumulated in most isolates suggested a limited circulation in humans. Only one SL-3 isolate contained seven mutations, which is compatible with more than half a year of circulation.

**Conclusion:**

Since OPV was withdrawn from the immunisation schedule in Poland in 2016, detection of SL-3 in airport sewage may indicate the events of importation from a region where OPV is still in use. Our study shows that environmental surveillance, including airport sewage investigation, has the capacity to detect emerging polioviruses and monitor potential exposure to poliovirus importation. Poliovirus detection in sewage samples indicates the need for sustaining a high level of polio immunisation coverage in the population.

## Introduction

A number of factors influence the spread of infectious diseases that are prone to cause epidemics. One of them is travel, which is strongly associated with importation of pathogens into a new geographical area, where they may spread in a susceptible population [1].

Polioviruses (*Picornaviridae* family) are human pathogens which may easily be imported via travellers. These small viruses with single-stranded positive-sense genomic RNA are highly resistant to chemical inactivation. Low sensitivity to environmental factors allows them to survive in the environment for a considerable period of time [2]. They are transmitted through the faecal-oral and oral-oral route and multiply in the gastrointestinal tract. Faecal viral shedding can continue for several weeks and occurs also in asymptomatic cases. The long period of virus excretion, high frequency of asymptomatic infections and large numbers of excreted poliovirus particles also affect the success of virus introduction into susceptible populations.

There are many examples of wild poliovirus (WPV) importation to previously polio-free countries. Some of them were related to detection of WPV in sewage samples without cases of acute flaccid paralysis and associated with the early detection of silent virus circulation as in Israel in 2013, and others were connected with a single virus detection in the sewage sample as in Egypt in 2008 and 2010 [3-5]. These cases prove that environmental poliovirus surveillance has the capacity to detect and monitor wild strains.

The last case of poliomyelitis caused by WPV in Poland was notified in 1984 [6], the last WPV infection in Europe was in 1998, and the World Health Organization (WHO) has declared Europe polio-free since 2002. However, the risk still exists of poliovirus reintroduction to Europe from endemic areas and countries with suboptimal polio vaccine coverage where the oral polio vaccine (OPV) is routinely used. Poliovirus outbreaks in Tajikistan (2010), Israel (2013) and Ukraine (2015) have demonstrated this possibility [5,7,8]. To achieve poliovirus eradication, sensitive tools are needed to monitor poliovirus transmission and international spread. One of them is environmental surveillance, which can detect introduction and silent circulation of polioviruses with even greater sensitivity than surveillance of acute flaccid paralysis.

The aim of this study was to apply environmental surveillance at an international airport in Poland to monitor the importation of polioviruses via international air transport.

## Methods

### Sewage samples

Samples of raw sewage were collected at the international airport in Warsaw, Poland – the Warsaw Chopin Airport – between July 2017 and March 2020. We collected 1,600 mL of raw sewage weekly using a grab method. A total of 142 sewage samples were processed according to the protocol described earlier [9]. In brief, AlCl_3_ (final concentration: 0.5 mM) was added to 1,600 mL of sewage sample, and the pH was adjusted to 3.5. Following addition of 800 µL of SiO_2_ slurry, the samples were stirred for 30 min, followed by centrifugation at room temperature and 1,500 × g for 5 min to pellet the SiO_2_. The virus was recovered by rocking the pellet for 20 min with 9.6 mL of 5 mM glycine (pH 9.5) containing 3% (w/v) beef extract. After centrifugation for 5 min at 4 °C and 1,500 × g, the supernatant was treated with chloroform (1:1) with rocking for 20 min. After a final centrifugation, the concentrates were used to inoculate cell cultures.

### Virus isolation and characterisation

Rhabdomyosarcoma (RD) and L20B cells were cultivated in minimal essential medium (MEM) supplemented with 10% fetal bovine serum (FBS). Virus isolation was based on WHO protocols and involved inoculating a sewage concentrate into RD and L20B cells, followed by a series of cell passages from the RD arm or the L20B arm, which can select for polioviruses in specimens. [10]. For each sample, 0.2 mL chloroform-extracted sewage concentrate was inoculated on eight RD cell culture tubes (RD arm) and eight L20B (L20B arm) cell culture tubes. Half of the tubes in each arm (n = 4) were incubated at 36 °C and the other half at 40 °C to identify isolates that had lost temperature sensitivity and reverted to a wild-type poliovirus phenotype. If a cytopathic effect (CPE) had not appeared after at least 5 days of observation, a blind passage was performed in the same cell line and examination was continued for a further 5 days. Three serial blind passages were carried out. If CPE appeared, a cross-passage was performed to the opposite cell line. Specimens with CPE in the RD arm or the L20B arm were then screened by realtime RT-PCR intratypic differentiation (ITD) assays according to WHO recommendations (Poliovirus realtime RT-PCR ITD 5.1 and vaccine-derived poliovirus (VDPV) 5.0 kits, produced by the United States Centers of Disease Control and Prevention). Samples demonstrating CPE only in RD cells in the RD arm were classified as non-polio enteroviruses (NPEV). All poliovirus isolates were sent to the WHO Regional Reference Laboratory at the Robert Koch Institute in Berlin, Germany for sequencing and confirmation of the ITD results. The isolates were analysed by sequencing of the entire VP1 genomic region and were identified based on their degree of genetic divergence from the parental Sabin strain [11]. Phylogenetic and molecular evolutionary analyses were conducted using MEGA version X [12]. A phylogenetic tree was computed using the neighbour-joining method with 1,000 bootstrap replicates. The sequences have been assigned GenBank accession numbers ON009832 to ON009839.

## Results

Between June 2017 and March 2020, we collected samples from the international airport of Warsaw with a frequency of once a week. In total, 142 samples were collected and analysed. Seven samples (4.9%) were positive for polioviruses after isolation in cell culture. The proportion of positive samples ranged from 0 of 26 in 2017 to five of 52 in 2019. We detected NPEV in 134 samples, with a positivity rate of 94.4% (134/142). The proportion of NPEV was the highest in 2019 and 2020 (100%) and the lowest in 2017 (84.6%). (Table 1).

**Table 1:** Proportion of poliovirus and non-polio enterovirus detections in airport sewage samples by cell culture isolation, Warsaw, Poland, July 2017–March 2020 (n = 142 samples)

Year of detection	Poliovirus			Non-polio enterovirus		
	Positive	Tested	%	Positive	Tested	%
2017	0	26	0.0	22	26	84.6
2018	1	53	1.9	49	53	92.5
2019	5	52	9.6	52	52	100
2020	1	11	9.1	11	11	100
**Total 2017–2020**	**7**	**142**	**4.9**	**134**	**142**	**94.4**

Three isolates were detected simultaneously in the RD and L20B arms, two in the RD arm and two in the L20B arm. One poliovirus isolation was possible at elevated temperature of isolation (40 °C). According to the algorithm for poliovirus isolation, all positive samples classified as poliovirus were characterised by realtime RT-PCR ITD assays as Sabin-like poliovirus type 3 (SL-3) (Table 2). No wild type or VDPV were found during the 3 years of the study.

**Table 2:** Poliovirus and non-polio enterovirus detected in cell culture and characterisation of isolates from airport sewage samples, Warsaw, Poland, July 2017–March 2020 (n = 7)

Sampling date	Number positive among four tubes tested per arm and temperature				ITD/VDPV results
	L20B arm, incubation at 36 °C	RD arm, incubation at 36 °C	L20B arm, incubation at 40 °C	RD arm, incubation at 40 °C	
31 Dec 2018	1	1	0	0	PV3-SL
15 Jan 2019	1	1	0	0	PV3-SL
22 Jan 2019	0	1	0	0	PV3-SL
26 Mar 2019	1	0	1	0	PV3-SL
4 Jun 2019	0	1	0	0	PV3-SL
6 Aug 2019	1	1	0	0	PV3-SL
14 Jan 2020	1	0	0	0	PV3-SL

ITD: intratypic differentiation; RD: rhabdomyosarcoma cells; VDPV: vaccine-derived poliovirus.

Seven isolates detected at 36 °C and one detected at 40 °C were characterised by sequencing. The isolates shared 99.22–99.78% nucleotide identity in the VP1 region with the parental Sabin strain type 3, indicating that these PV strains had originated from OPV (> 99% VP1 sequence identity). The number of nucleotide substitutions in the VP1 coding region varied from two to seven (0.22–0.78%). More than half (9/17) were non-synonymous substitutions (Table 3). All isolates were classified as Sabin-like strains according to the WHO definition (≤ 9 nucleotide substitutions for type 3). One isolate collected in January 2019 had seven substitutions (0.78%) and was characterised according to the classification used previously by other authors [13] as high-mutant strain pre-VDPV (between six and nine nucleotide sequence variations). The low extent of sequence divergence suggests that these viruses were probably associated with a recent vaccination event. All isolates showed a single point mutation at nucleotide position 17 (C > U).

**Table 3:** Molecular characterisation of poliovirus SL-3 isolates from airport sewage based on the VP1 sequences, Warsaw, Poland, July 2017–March 2020 (n = 8)

SL-3 isolates	Nucleotide position on VP1 sequence with reference to the complete genome sequence of the reference Sabin 3 strain (AY184221)																	Nucleotide substitutions		VP-1 sequence results
	2493	2636	2665	2762	2806	2905	2975	2989	3028	3178	3184	3228	3248	3283	3305	3335	3338	n	%	
Sabin 3	C	G	C	A	C	U	U	C	C	U	U	A	A	C	C	A	A			
31 Dec 2018	**U**	-	-	-	T	C	-	-	-	-	-	-	-	-	-	-	-	3	0.33	SL
15 Jan 2019	**U**	**A**	-	-	-	-	-	-	-	-	-	**G**	**G**	-	-	-	-	4	0.44	SL
22 Jan 2019	**U**	**A**	-	-	-	-	-	-	U	-	-	**G**	**G**	-	**U**	**G**	**-**	**7**	**0.78**	preVDPV
26 Mar 2019 (36 °C)	**U**	-	-	-	-	-	-	-	-	C	C	-	-	U	-	-	-	4	0.44	SL
26 Mar 2019 (40 °C)	**U**	-	-	-	-	-	**A**	-	-	C	-	-	-	U	-	-	-	4	0.44	SL
4 Jun 2019	**U**	-	U	-	-	-	-	-	-	-	-	-	-	-	-	-	-	2	0.22	SL
6 Aug2019	**U**	-	-	-	-	-	-	-	-	-	-	-	**G**	-	-	-	-	2	0.22	SL
14 Jan2020	**U**	-	-	**G**	-	-	-	U	-	-	-	-	-	-	-	-	**G**	4	0.44	SL

SL: Sabin-like.

Bold letters and grey shading: non-synonymous substitutions. Hyphens: identity with the reference Sabin 3 strain (AY184221).

The complete VP1 protein sequences differed by between 0.33% and 2% among the sewage SL-3 isolates. A mutation in amino acid VP1–6 (T > I) was a direct reversion to the parental Leon sequence. Mutations in amino acids VP1–96, VP1–287, VP1–288 were located at neutralisation antigenic sites NAg1 and NAg3a (Table 4).

**Table 4:** Amino acid changes in VP1 protein between SL-3 isolates from airport sewage, Warsaw, Poland, July 2017–March 2020 (n = 8)

SL-3 isolates	Amino acid position on VP1 sequence in relation to the reference Sabin 3 (AY184221)									Amino acid substitutions	
	6	54	96	167	251	258	277	287	288	n	%
Sabin 3	T	A	T	S	K	I	P	N	N		
31 Dec 2018	I	-	-	-	-	-	-	-	-	1	0.33
15 Jan 2019	I	T	-	-	R	V	-	-	-	4	1.33
22 Jan 2019	I	T	-	-	R	V	S	**D**	-	**6**	**2.00**
26 Mar2019 (36 °C)	I	-	-	-	-	-	-	-	-	1	0.33
26 Mar 2019 (40 °C)	I	-	-	T	-	-	-	-	-	2	0.66
4 Jun 2019	I	-	-	-	-	-	-	-	-	1	0.33
6 Aug 2019	I	-	-	-	-	V	-	-	-	2	0.66
14 Jan 2020	I	-	**A**	-	-	-	-	-	**D**	3	1.00

SL: Sabin-like.

Bold letters and grey shading: amino acid changes in the neutralisation antigenic site 1 (NAg1) (VP1: 89-100) and NAg3a (VP1: 286-290). Hyphens: identity with the reference Sabin 3 strain (AY184221).

Analysis of the relative genetic relationships of the sewage SL-3 showed that most of the isolates had acquired genetic changes independently, with the exception of three SL-3 from 2019 (two from January and one from August) and two isolates from March 2019 (found in the same sewage sample collected on 26 March 2019) (Figure).

**Figure:**
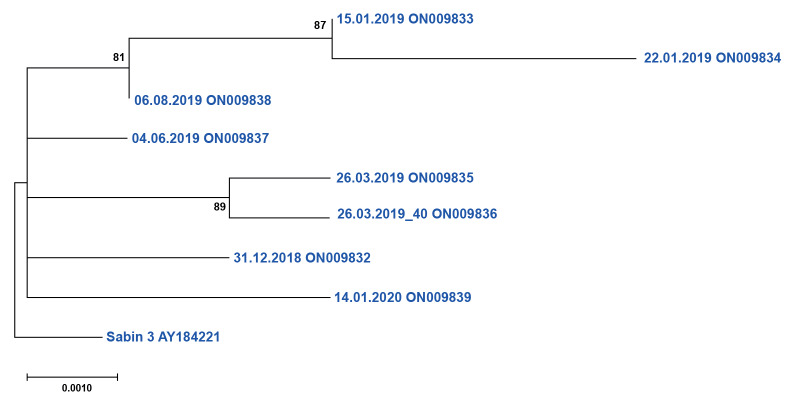
Phylogenetic tree depicting the relative genetic relationships of poliovirus SL-3 airport isolates supplemented with the sequence of Sabin 3, Warsaw, Poland, July 2017–March 2020 (n = 8)

The tree was constructed from nucleotide sequences using neighbour-joining algorithm with Kimura 2-parameter substitution model.

## Discussion

Environmental surveillance is an important additional part of the strategy of the Global Polio Eradication Initiative. It is a very sensitive method for PV detection. Theoretically, it is capable of detecting one infected individual among 10,000 uninfected ones [14]. Its effectiveness as a tool for monitoring of silent poliovirus circulation in the population has been confirmed repeatedly [4,15,16]. Several countries in Europe currently use regular environmental surveillance as a supplement to clinical surveillance [15,17-20]. The European activities in this area have led to the detection of VDPV-infected persons in the community, the incidental identification of SL strains in countries using inactivated poliovirus vaccine (IPV) and the confirmation of polio-free status. Between July 2017 and March 2020, we detected polioviruses in seven samples (4.9%) from the Warsaw airport surveillance programme, all of which were characterised as Sabin-like vaccine strains. No WPV or VDPV were detected during the study period. Detection of Sabin and Sabin-like viruses is common following vaccination with OPV. The poliovirus detection rate in sewage samples in OPV-using countries ranged from 9% (Russia 2004–2017) to 82.1% (China 2013) and depends on the methodology of monitoring and an ongoing vaccination campaign; also climate can be a factor [21,22]. An earlier Polish study, conducted during the period of OPV use, showed an occurrence of Sabin-like strains in 18.8% of tested sewage samples [23]. Since 2016, Poland has used only IPV in vaccination campaigns after OPV was withdrawn from the national immunisation schedule. It is believed that the circulation of vaccine strains should stop 3 months after OPV withdrawal. The experiences of countries using solely IPV showed occasional detection of Sabin-like strains in sewage samples, probably connected with visiting individuals immunised with OPV abroad, suggesting poliovirus importation [15,17-20,24]. Many countries in the WHO European Region still used OPV after switching from the trivalent to the bivalent vaccine in 2016, therefore detection of Sabin-like strains in Europe should not be surprising. After changes in the rules of employing foreign citizens in 2018, migrant workers coming to Poland from Ukraine, estimated at 1–2 million, could have influenced the result obtained in our project. Ukrainian citizens were the fifth largest group of passengers served at Polish airports in 2019 [25].

A detection of poliovirus in a sewage sample represents recent importation of the virus or circulation in the community. However, a detection of poliovirus in airport sewage samples rather points to poliovirus importation. The airport is subject to a large flow of people from around the world (over 50 million passengers were handled at the Warsaw Chopin Airport in 2017 to 2020) but, besides the travellers, also airport employees are part of the airport sampling site (they consisted of less than 5% of the daily number of passengers at the Chopin Airport). This should also be taken into account when interpreting the results; for this reason, some virus detections may be related to the local community.

According to the WHO, individual importations of polioviruses are highly unlikely to be detected by environmental surveillance and the repeated detection of virus in a sampling site almost guarantees that virus is circulating in the population, but it should be taken into account that this opinion concerns regular monitoring in the local community [14]. When detecting high-risk polioviruses (WPV and VDPV) in sewage from the airport site, supplemental strategies should be considered. In that case, increasing the frequency of specimen collection is not as relevant as expanding environmental surveillance to the local community and enhancing clinical surveillance. In Poland, the activities after detection of poliovirus are regulated by the National Plan for a polio event or outbreak response from 2019. The obtained results in this project prompted us to expand environmental surveillance to the entire area of Warsaw.

The SL-3 viruses isolated in wastewater samples at Warsaw airport showed little genetic divergence from the Sabin strain. The number of mutations accumulated in most airport SL-3 isolates suggests limited circulation of the viruses in humans. Only in one SL-3 from January 2019 we found seven mutations (0.77%), which is compatible with more than half a year of circulation (assuming a substitution rate of 1% per year). All detected SL-3 isolates were mutated at VP1–6 (T > I) but this replacement is described as one of the mutations of a sub-strain of Sabin 3 used in vaccine manufacturing [26]. On the other hand, three other mutations we identified which are located in the neutralisation antigenic sites may be connected with antigenic drift and immune escape. These changes may have epidemiological consequences, contributing to enhanced viral transmissibility and transformation to epidemic strains [27].

In a population with suboptimal immunisation coverage, OPV strains may revert to neurovirulent form as a VDPV, as it happened in Ukraine in 2015 and 2016 [8]. Two independent detections of cVDPV1 in paralytic cases in Zakarpattia in Ukraine indicated a need to maintain high poliovirus immunisation coverage in all European countries and showed a possibility of poliovirus reintroduction in Europe. Only populations protected by vaccines resist introduction. Considering the growing activity of anti-vaccination movements in Europe, such a possibility is increasing. In Poland, the polio immunisation coverage is not optimal (85.5% in 2020) and has a downward trend (91.6% in 2016, 90.1% in 2017, 87.4% in 2018 and 86.6% in 2019), which may risk the country's polio-free status. The activity of anti-vaccination movements has led to a noticeable increase in the number of parents refusing vaccination in Poland, from 3,000 in 2008 to 48,000 in 2019 [28]. Therefore, achieving and sustaining a polio-free status will require maintaining strategies that allow rapid detection and control of outbreaks following emergence of VDPV or potential reintroduction of WPV. Environmental surveillance might play a substantial role in providing evidence for the certification of polio-free status.

The proportion of NPEV positive sewage samples is important when assessing the quality of the applied environmental surveillance. According to WHO, at least 30% of concentrated sewage from grab samples should reveal NPEV [14]. The detection of non-polio enteroviruses in the airport samples was very high (over 90%), which might indicate high effectiveness of virus recovery. Considering this very high NPEV detection rate and that OPV is not used in Poland, we concluded that the setup of our airport surveillance is sensitive enough to detect low concentrations of poliovirus. Airport sewage monitoring can provide important information about the exposure to poliovirus importation, but their effectiveness depends substantially on the methodology of monitoring.

The present study has several limitations. On the one hand, it is possible to monitor a large group of airport passengers with sewage samples, but on the other hand, their true number and place of residence are unknown, which prevents identification of individuals, pinpointing their origin and conducting further epidemiological investigations. Therefore, the detection of polioviruses in airport sewage can be used solely as an alert that requires supplemental strategies. Poland was not conducting the national environmental surveillance during the period of this project and performed solely AFP surveillance, which made it impossible to assess our results in the context of other epidemiological information. In addition, the representativeness of the tested samples may have been affected by the fact that the study only included samples from the airport terminal itself and not samples from airplanes, which dispose of their waste separately. This means that tested samples were deprived of some material related to air transport in Warsaw. Of note, the study was stopped at the end of March 2020, when air traffic restrictions were introduced during the COVID-19 pandemic.

Another limitation of the study is the research design and used method. According to the WHO recommendations, at least 3 mL of sewage concentrate should be inoculated into five L20B flasks and one RD culture flask for virus isolation. The present study used more than 3 mL of sewage concentrate for virus isolation, but the proportion of used RD and L20B cultures was the same, which could affect the sensitivity and high NPEV rate. Moreover, the goal of monitoring the importation of WPV was also to achieve because the Chopin airport does not have direct flights to regions endemic for WPV (Afghanistan, Pakistan).

## Conclusions

This study showed that environmental surveillance, including investigation of sewage from international airports, has the capacity to detect emerging poliovirus strains and monitor exposure to poliovirus importation. Since OPV was withdrawn from the national immunisation schedule in Poland in April 2016, detection of SL-3 strains in airport sewage may indicate importations from regions where OPV is still in use. Each poliovirus importation to a polio-free country indicates the need to sustain a high level of polio immunisation coverage in the population.
